# The usefulness of [18F]FDG-PET/CT in detecting and managing cancers with unknown primary site depends on histological subtype

**DOI:** 10.1038/s41598-021-96451-z

**Published:** 2021-09-06

**Authors:** Ella Nissan, Uri Amit, Leo Baron, Amit Zabatani, Damien Urban, Iris Barshack, Tima Davidson

**Affiliations:** 1 Sackler Faculty of Medicine, Tel Aviv University, Tel Aviv, Israel; 2 Radiation Oncology Department, Chaim Sheba Medical Center, Tel Hashomer, Israel; 3 The Dr. Pinchas Borenstein Talpiot Medical Leadership Program, Chaim Sheba Medical Center, Tel Hashomer, Israel; 4 Department of Orthopedic Surgery, Chaim Sheba Medical Center, Tel Hashomer, Israel; 5 Department of Oncology, Chaim Sheba Medical Center, Tel Hashomer, Israel; 6 Department of Pathology, Chaim Sheba Medical Center, Tel Hashomer, Israel; 7 Department of Nuclear Medicine, Chaim Sheba Medical Center, Tel Hashomer, Israel

**Keywords:** Cancer imaging, Cancer of unknown primary

## Abstract

We assessed the role of [18F]FDG-PET/CT in identifying and managing cancer of unknown primary site (CUP syndrome). We reviewed [18F]FDG-PET/CT scans of individuals with CUP syndrome recorded in clinical referral letters from 2012 to 2019. We evaluated the identification of primary tumor (PT) by [18F]FDG-PET/CT, according to histological subtype, and the impact on clinical management. The median age was 65 years, 36/64 males (56%). PTs were detected in 28/64 (44%) patients. Detection was significantly lower in patients with squamous cell carcinoma (SCC) than with other histologies combined, p = 0.034. Mean age, mean SUVmax (10.6 ± 6.0) and organ involvement were similar between patients with and without discovered PTs; and between patients with SCC and with other histologies combined. However, those with SCC were less likely than the others to present with multi-lesion involvement, p < 0.001. [18F]FDG-PET/CT interpretations apparently affected treatment of 8/28 (29%) patients with PT detected, and in none of the 35 whose PT was not discovered, p < 0.001. [18F]FDG-PET/CT appeared helpful in detecting PT in almost half the patients with CUP syndrome; the lowest rate was for patients with SCC pathology. PET/CT showed limited overall value in guiding clinical management, however benefited those with discovered PT.

## Introduction

Cancer of unknown primary site (CUP syndrome) is a diverse group of cancers in which the anatomical site of origin remains occult despite detailed investigations. CUP syndrome accounts for 2–5% of cancers worldwide^1,2^. The median age at presentation is 60–65 years and diagnosis is more common in men than women by a ratio of 3:2^3^. CUP syndrome has a wide variety of clinical presentations and many histological types. Sensitivity to treatment tends to be low and median survival time is 6–10 months^4^. Due to the difficulty of diagnosis and lengthy investigations, time from initial presentation to treatment is longer and pretreatment costs are higher in patients with CUP syndrome than in patients whose primary tumor site is known^5^.

CT and conventional MRI enable the detection of only 22–36% of the primary sites of CUP syndrome^1,6^. These low detection rates have been attributed to functional limitations of these imaging modalities. Both CT and MRI enable the detection of anatomical abnormalities and abnormal contrast enhancement; however, small and non-enhancing lesions in normal sized structures may be missed.

In contrast to conventional imaging modalities, F18-fluorodeoxyglucose PET/CT ([18F]FDG-PET/CT) does not have the abovementioned drawbacks as it leverages the increased glucose metabolism in many malignant cancers (Warburg effect) to detect abnormal uptake of the [18F]FDG^7^. However, in a head and neck area, CT/MRI is still a better diagnostic tool in assessing abnormalities than other modalities^8,9^. [18F]FDG-PET/CT is commonly used to search for occult primary tumors undetected by conventional diagnostic methods^8,9^. While the use of [18F]FDG-PET/CT in the detection of primary tumors (PTs) in CUP syndrome has been suggested for at least two decades^3,10,11^, its roles in the diagnostic workup of patients with disseminated CUP syndrome remain inconclusive^12^.

The primary purpose of this study was to assess the role of [18F]FDG-PET/CT in the identification of PTs, and therefore in the management of CUP syndrome in patients with negative conventional diagnostic workup. The secondary objectives were to evaluate the ability of [18F]FDG-PET/CT in discovering PTs according to their histological subtypes, and thus to evaluate the impact on clinical management.

## Results

Of 33,679 [18F]FDG-PET/CT scans performed during the study period, 64 [18F]FDG-PET/CT included the term “Unknown Primary”. Demographic and clinical characteristics of the population are presented in Table 1. The study cohort comprised 36 males and 28 females; the median age was 65 years (range: 18–87). The patients had a total number of 145 [18F]FDG–avid lesion sites, with a mean SUVmax of 10.6 (range: 2.2–27.8). The most common sites of [18F]FDG uptake were the lymph nodes: 39/145 (27%), bones: 24 (17%), liver: 17 (12%), lungs: 17 (12%), regions of the head and neck: 7(5%) and brain: 6 (4%). The remaining 35 sites (23%) included the uro-gynecological system, esophagus, peritoneum, skin, colon, thyroid and muscles. The median number of sites/organs involved simultaneously in the same patient was 2 (range: 1–5), as follows: 20 patients (31%) had a finding in one site, 26 (41%) had findings in two sites, 11 (17%) in 3 sites, 5 (8%) in 4 sites, and 2 (3%) had findings in more than five sites. Additionally, most patients, 47/64 (73%), had a multi-lesion metastatic spread disease.

**Table 1:** Demographic, clinical and radiographic characteristics of the study cohort.

	Patients (n = 64)
**Sex**	
Male Female	36 (56%) 28 (44%)
Median age, years	65 (range 18–87)
Total [18F]FDG-avid lesions	145
Mean SUVmax, (standard deviation)	10.6 (6)
**Rate of organ/sites of 145 [18F]FDG-avid lesions**^**a**^	
Lymph nodes Bones Liver Lungs Head and neck Brain Other^b^	39 (27%) 24 (16%) 17 (11%) 17 (11%) 7 (5%) 6 (4%) 35 (23%)
**Number of uptake organ/sites**	
1 2 3 4 5 or more	20 (31%) 26 (416%) 11 (17%) 5 (8%) 2 (3%)
**Tumor histology**	
Origin was pathologically and immunohistochemically suggested Poorly differentiated carcinoma Squamous cell carcinoma Adenocarcinoma Neuroendocrine carcinoma	30 (47%) 14 (22%) 10 (16%) 9 (14%) 1 (2%)
**Primary lesion detected by [18F]FDG-PET/CT**	
Detected Undetected	28 (44%) 36 (56%)
**Patient management before [18F]FDG-PET/CT scan**^**c**^	
Specific chemotherapy Empiric chemotherapy Palliative radiation Surgery Chemoradiation No medical treatment	31 (48%) 4 (6%) 6 (9%) 5 (8%) 6 (9%) 11 (17%)

^a^The total number of sites exceeds 100% due to the involvement of more than one site in some patients.

^b^‘Other’ includes the uro-gynecological system, esophagus, peritoneum, skin, colon, thyroid and muscles.

^c^Number of patients with management data is 63, one patient with squamous cell carcinoma and an undetected primary site was lost to follow-up.

Tumor histologies included: 14 (22%) poorly/undifferentiated carcinomas, 10 (16%) squamous cell carcinoma (SCC), 9 (14%) adenocarcinomas, 1 (2%) neuroendocrine carcinoma and 30 (47%) tumors for which the origin was pathologically and immunohistochemically suggested (including: melanoma, thyroid, peritoneal mesothelioma, sarcoma, thyroid carcinoma, thymoma, ovarian carcinoma, gastrointestinal tumor, pancreatic and biliary carcinoma, carcinoma of breast and lung).

### Primary tumors (PTs) discovered by [18F]FDG-PET/CT

**Figure 1:**
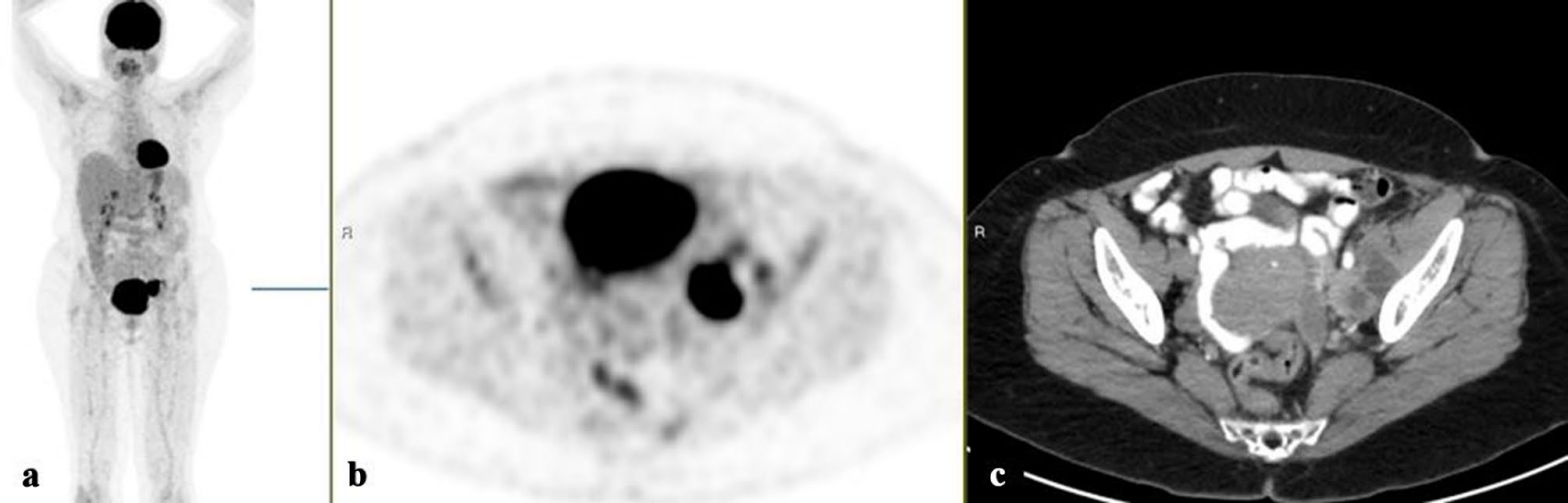
[18F]FDG-PET/CT: maximum intensity projection (MIP), **(a)** representative PET **(b)** and CT **(c)** axial slices. A 65-year-old woman with a biopsy-proven squamous cell carcinoma from a pelvic mass (A. b). Left obturator [18F]FDG avid enlarged lymph nodes.

**Figure 2:**
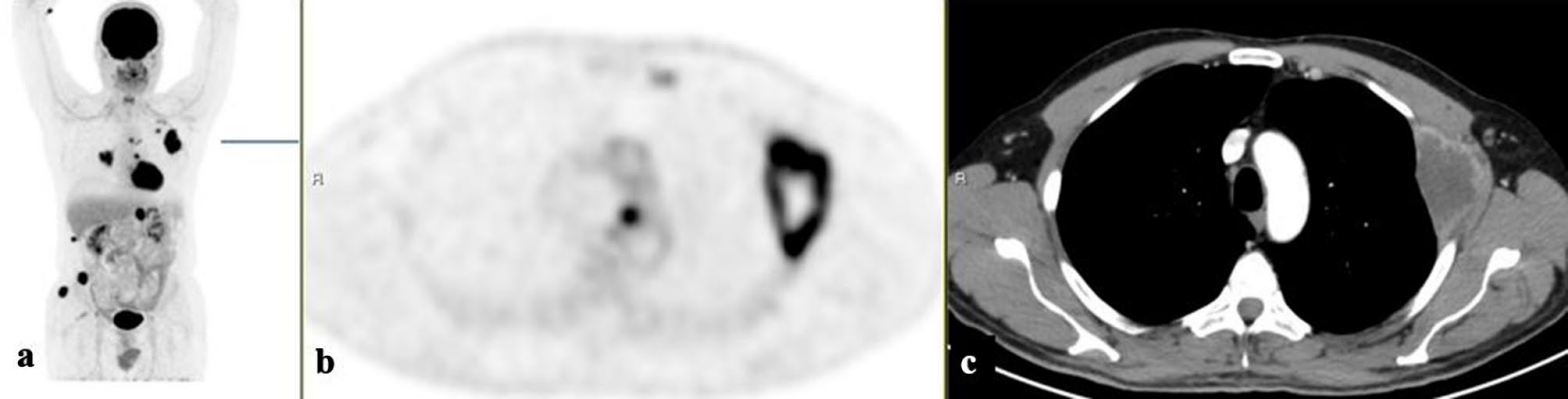
[18F]FDG-PET/CT: maximum intensity projection (MIP), **(a)** representative PET **(b)** and CT **(c)** axial slices. A 49-year-old man with a biopsy-proven poorly-differentiated adenocarcinoma from a chest wall mass (A. b). Multiple sites of [18F]FDG avid lesions in the bones and in the left adrenal.

**Figure 3:**
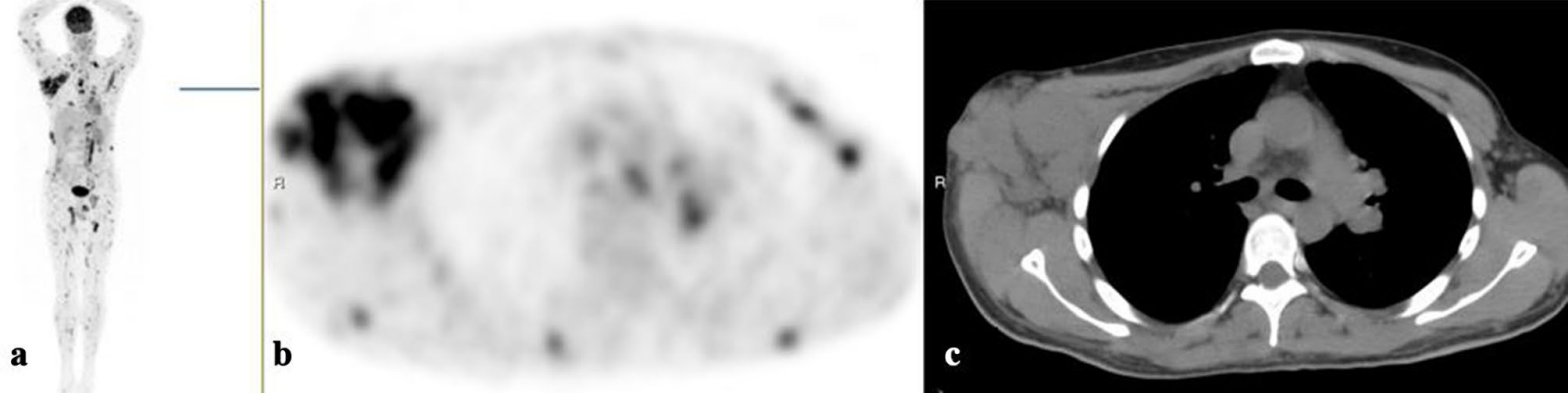
[18F]FDG-PET/CT: maximum intensity projection (MIP), **(a)** representative PET **(b)** and CT **(c)** axial slices. A 35-year-old man with melanoma diagnosed by a biopsy taken from the right axillary lymph nodes (A. b). Large lymph nodes in the right axilla and multiple [18F]FDG-avid soft tissue lesions.

[18F]FDG-PET/CT discovered the PT in 28 patients (44%); while in the remaining 36 (56%), the PTs were not located. Table 2 presents the characteristics of the two groups. Differences in mean patient age) 61.2 vs. 61.9 years, p = 0.72), mean SUVmax of the lesions (10.3 vs 10.8, p = 0.08) and the mean number of organs/sites involved by [18F]FDG-avid lesions (2.4 vs. 1.8, p = 0.47) were not statistically different between patients with and without an identified PT. Variations in anatomic organs and sites involved by [18F]FDG-avid metastatic lesions were also similar between patients with and without discovered PTs. The rates of identification of PTs relative to the histologic groups were: 18/30 (60%) of patients with tumors for which the origin was pathologically and immunohistochemically suggested, 4/9 (44%) of adenocarcinomas, 4/14 (29%) of poorly differentiated carcinomas, 1/10 (10%) of SCC and the sole (100%) neuroendocrine carcinoma. [18F]FDG-PET/CT detection of PTs was significantly worse for the patients with SCC than for the 54 patients with other pathologies, considered as a combined group: 1/10 (10%) vs. 27/54 (50%), p = 0.03. The only patient in whom PT was detected was one of 7 (14.3%) patients with SCC with head and neck findings. Therefore, we decided to examine in more depth the subgroup of patients with SCC pathology and to compare their characteristics to the combined subgroup of patients with pathologies other than SCC (Table 3). Statistically significant differences were not observed between these two subgroups of patients, in mean age (61.7 vs 61.6 years, p = 0.85), mean SUVmax of [18F]FDG -avid lesions (11.3 vs 10.4, p = 0.38) and the mean number of involved organs/sites (1.5 vs 2.2, p = 0.09). Variations in anatomic organs and locations involved by [18F]FDG-avid metastatic lesions were similar between patients with SCC and those with the other histologies combined. However, among the patients with SCC, the proportion with multi-lesion spread was substantially lower than for the rest of the cohort: 2/10 (25%) vs. 45/54 (83%), p < 0.001, see Figs. 1, 2 and 3.

**Table 2:** Characteristics of patients with cancer of unknown primary site according to [18F]FDG-PET/CT detection of the primary site.

	Tumor detection by [18F]FDG-PET/CT		P value
	Detected (n = 28)	Undetected (n = 36)	
Mean age	61.2 (SD ± 13.6)	61.9 (SD ± 14.7)	0.72
Mean SUVmax	10.3 (SD ± 5.0)	10.8 (SD ± 6.8)	0.08
Mean number of organ/sites involved	2.4 (SD ± 1.0)	1.8 (SD ± 1.0)	0.47
**Histology of the revealed primary tumors**			
Origin was pathologically and immunohistochemically suggested Adenocarcinomas Poorly differentiated carcinomas Squamous cell carcinoma Neuroendocrine carcinoma	18/30 (60%) 4/9 (44%) 4/14 (29%) 1/10 (10%) 1/1 (100%)		
**[18F]FDG-PET/CT effects on management**^**a**^			< 0.001
Changed Unchanged	8 (29%) 20 (71%)	0 35 (100%)	

^a^The number of patients with management data is 63, one patient with squamous cell carcinoma and an undetected primary site was lost to follow-up.

**Table 3:** Characteristics of patients with cancer of unknown primary site according to tumor histology: squamous cell carcinoma versus all other tumor histologies.

	Tumor histology		P value
	Squamous cell carcinoma (n = 10)	Other (n = 54)	
Mean age	61.7 (SD ± 13.5)	61.6 (SD ± 14.4)	0.85
Mean SUVmax	11.3 (SD ± 7.4)	10.4 (SD ± 5.8)	0.38
Mean number of sites involved	1.5 (SD ± 0.5)	2.2 (SD ± 1.0)	0.09
**Primary lesion detected by [18F]FDG-PET/CT**			0.03
Detected Undetected	1 (10%) 9 (90%)	27 (50%) 27 (50%)	
**Number of lesions**			< 0.001
2 or less Above 2	8 (80%) 2 (20%)	9 (17%) 45 (83%)	
**[18F]FDG uptake organ/sites**			
Lymph nodes Bones Liver Lungs Brain Head and neck Other^a^	7 0 0 0 0 2 2		
**[18F]FDG-PET/CT effects on management**^**b**^			1.0
Changed Unchanged	1 (11.1%) 8 (88.9%)	7 (13%) 47 (87%)	

^a^‘Other’ includes the uro-gynecological system, esophagus, peritoneum, skin, colon, thyroid and muscles.

^b^The number of patients with management data is 63, one patient with squamous cell carcinoma and an undetected primary site was lost to follow-up.

### [18F]FDG-PET/CT and treatment management

Clinical data regarding treatment were available for 63 patients; of them, 31 (48%) received specific chemotherapy, 4 (6%) empiric chemotherapy and 6 (9%) palliative radiation; 5 (8%) underwent surgery and 6 (9%) chemo radiation. Eleven (17%) received best supportive care, because of their poor performance status and clinical situation. One patient with SCC histology, whose PT was not detected by PET/CT, was lost to follow up.

The [18F]FDG-PET/CT findings did not appear to affect the clinical management of 55 (87%) of the 63 patients with available data. Treatment was apparently affected in 8/28 (29%) patients with a PT detected by [18F]FDG-PET/CT: seven received chemotherapy that was specific to the diagnosis, and one patient received palliative radiotherapy.

Treatment was apparently not affected by the [18F]FDG-PET/CT scan in any of the 35 for whom the PT was not detected (p < 0.001, Table 2). Therefore, considering the entire cohort, [18F]FDG-PET/CT findings seem to have changed clinical management in 8/63 (13%) patients. Despite the much lower detection rates among patients with SCC, the effect of [18F]FDG-PET/CT findings on clinical management did not appear to differ between these patients and those with other pathologies combined: 1/9 (11%) vs. 7/54 (13%) (p = 1.0, Table 3).

Since tumors for which the origin was pathologically and immunohistochemically suggested comprised the largest subgroup of our cohort, 30 (47%), we compared changes in management between this subgroup of patients and all the other patients combined without a pathologically and immunohistochemically suggested origin. We found similar rates of treatment changes following [18F]FDG-PET/CT for the two subgroups: 4/30 (13%) and 4/33 (12%), respectively.

[18F]FDG-PET/CT detected a higher proportion of PTs among patients with tumors for which the origin was pathologically and immunohistochemically suggested than among all the other patients combined: 18/30 (60%) vs. 10/34 (30%), p = 0.014. However, detection of PTs affected treatment in a smaller proportion of the patients of the former than the latter, 4/18 (22%) vs. 4/10 (40%).

## Discussion

More than one decade ago, a multidisciplinary expert panel of oncologists, radiologists and nuclear physicians recommended the use of [18F]FDG PET in the diagnosis of patients with CUP syndrome^13^. Despite the common use of this imaging technique in this context, data are sparse regarding the characteristics of CUP syndrome for which [18F]FDG-PET/CT is most and least effective. Interestingly, in the current study of patients with CUP syndrome and negative conventional imaging, [18F]FDG-PET/CT detected the PT in only 1 (10%) of the patients with SCC compared to 50% of all those with other pathologies. Nonetheless, the apparent effects of the [18F]FDG-PET/CT findings on clinical management were similar between these two groups: 11% vs. 13%. Thus, surprisingly, the greater detection of PTs in pathologies other than SCC compared to SCC did not have clinical implications.

Our overall rate of tumor detection was 44%, which is within the range of 10%-75% reported in other studies^3,11,14–18^. While CUP syndrome is a relatively common clinical entity, presentations and histologies are diverse^9,19,20^ . Notably, consensus has not been reached as to whether CUP syndrome is simply a group of metastatic tumors with an undetected source, or a distinct entity with its own characteristics and behavior^17,21,22^. Most researchers currently believe that CUP syndrome is a heterogeneous collection of metastatic tumors^23^. Accordingly, treatment strategies have shifted from empiric cytotoxic therapies to identifying the PT and targeting therapy at the tumor type^24^. Importantly, detecting PT sites and additional metastases improves disease staging; this helps define prognosis and can better guide surgical intervention with curative intent^15^. Indeed, several studies have shown longer survival times in patients with CUP syndrome in whom a PT was detected^25,26^.

Sixteen percent of the patients in the current cohort were with SCC. This is higher than the 5% rate of CUP syndrome that was reported in a number of publications^19,27^, but substantially lower than the 57% rate that was reported in another study^11^. Among the reasons for the wide discrepancy in rates are the lack of a standardized definition of CUP syndrome, including the clinical workup and imaging tests required for the diagnosis^3,28^, and the resultant heterogeneity in selection criteria. Of our 10 patients with SCC, 7 (70%) had [18F]FDG-PET/CT uptake in the head and neck. Similarly, head and neck cancers were reported to represent 75% of patients with CUP syndrome and SCC histology^6^. In our series, the PT was detected in one of 7 (14.3%) of our patients with SCC who had head and neck findings. Similarly, Majchrzak et al. reported detection of PT in 17% (7/41) of patients with CUP syndrome of cervical lymph nodes with SCC metastases^29^. This compares with the detection by [18F]FDG-PET/CT of PTs that were not detected by other modalities in 25% of the patients with headand neck metastases in another cohort^30^. Notably, despite our relatively high proportion of patients with SCC, the age and sex distributions were comparable to those reported in other studies of [18F]FDG-PET/CT in CUP syndrome^3^. Further, patients’ age, SUVmax of the lesions, and site distribution of [18F]FDG-avid lesions were similar between patients whose PT was and was not detected by [18F]FDG-PET/CT; and between patients with SCC and those with all other pathologies combined. Thus, the distributions of age, involved organ/site and [18F]FDG avidity do not explain the low detection of PTs among our patients with SCC compared to those with other pathologies. Interestingly, our patients with SCC tumors were significantly more likely to present with limited-lesion metastatic spread disease involvement than were patients with the other pathologies combined. We speculate that this finding is due to lower metastatic rates in SCC or to poor [18F]FDG-PET/CT uptake in small SCC metastases, or to a combination of the two. SCC was shown to have a lower ratio of metastases per PT than adenocarcinoma^31^, while [18F]FDG-PET/CT uptake in SCC was shown to be directly correlated to tumor size, and lower in metastatic tumors than in PTs^32^.

Changes in treatment were attributed to [18F]FDG-PET/CT detection of primary sites in 29% of our patients with a newly detected PT. However, considering the entire cohort, including patients for whom the PT was not detected, [18F]FDG-PET/CT apparently affected clinical management in only 13%. This is on the lower end of the range of 10–58% (mean 35%) that was reported in a review of 10 studies^15^. That review found that patients with a planned curative treatment for cancers such as breast, ovary and prostate most benefited from the [18F]FDG-PET/CT scan; thus, differences between studies in the types of cancers may explain the large variability in detection rates^15^.

While its impact on clinical management may be limited to a subgroup of patients with discovered PT, [18F]FDG-PET/CT may have additional benefits for patients with CUP syndrome. This may explain disparities between studies in the interpretation of the usefulness of [18F]FDG-PET/CT for clinical decisions. Notably, Reinert et al.^11^ reported a PT detection rate in only 23% of patients with CUP syndrome, but changes in treatment management in twice the number of patients^11^. [18F]FDG-PET/CT has been recommended for accurate staging, monitoring of treatment response and follow-up in patients with CUP syndrome who undergo active therapy; and as an alternative to contrast CT in patients with severe iodine dye allergy^12^. Moreover, the use of [18F]FDG-PEFT/CT in place of conventional imaging may lead to earlier diagnosis of the PT and thus facilitate earlier targeted therapy^15^.

We acknowledge several limitations to this retrospective study. Our database search relied on proper documentation of the disease in the [18F]FDG-PET/CT reports and could therefore present an incomplete sample of patients from our institution. We did not have data regarding the workups that patients underwent according to their clinical presentations. Larger studies of patients with various pathologies and tumor sites are needed to better define the role of [18F]FDG-PET/CT in CUP syndrome and to identify the CUP syndrome subtypes whose management is most influenced by [18F]FDG-PET/CT results.

## Conclusion

[18F]FDG-PET/CT appeared helpful in detecting PT in almost half the patients with CUP syndrome; the lowest rate was for patients with SCC pathology. [18F]FDG-PET/CT showed limited overall value in guiding clinical management, however benefited those with discovered PT.

## Materials and methods

### Study design

We searched the Sheba Medical Center computerized database for [18F]FDG-PET/CT studies that included the term "Unknown Primary" in reports (in the graph of “indication” for the referral) recorded from April 2012 through February 2019. Medical history and tumor histopathology analysis were included in the clinical data. Imaging data were provided from the picture archive and communication system (PACS, Carestream Health 11.0, Rochester, NY), and clinical data from the computerized medical records at Sheba Medical Center.

The study inclusion criterion was an unknown PT according to the clinical referral letter at the performance of the [18F]FDG-PET/CT scan, with or without known tumor histology. For all the patients, the following were performed before [18F]FDG-PET/CT examination: a whole diagnostic workup including a physical examination, CT/MRI or US, and rhino-laryngoscopy in patients with cervical CUP syndrome. Pathological evaluation included immunohistochemical staining for tumor origin.

For this research, tumors were categorized into broad groups based on their histology type: adenocarcinoma, squamous cell carcinoma (SCC), poorly/undifferentiated carcinoma, neuroendocrine carcinoma and tumors in which the origin was pathologically and immunohistochemically suggested. The latter group was relevant when histology results from one of the metastatic lesions indicated a certain tumor origin (including: melanoma, thyroid, peritoneal mesothelioma, sarcoma, thyroid carcinoma, thymoma, ovarian carcinoma, gastrointestinal tumor, pancreatic and biliary carcinoma, carcinoma of breast and lung); however, prior conventional workup did not reveal the primary tumor site. Patients without any available histological data were not included in the study.

Metastatic spread was characterized by variations in anatomic organs or sites involved by [18F]FDG-avid metastatic lesions. Additionally, metastatic spread was evaluated according to the number of [18F]FDG-avid metastatic lesions and classified as a limited-lesion (up to two lesions) or as multi-lesion spread (more than two lesions).

### Image assessment

An experienced physician with two specializations (nuclear medicine and radiology) reviewed all the scans of all the patients included in the study. The intensity of [18F]FDG uptake in the lesions was calculated by standardized uptake values max (SUVmax), by manually generating a region of interest over the pathological lesion. The protocol of the [18F]FDG-PET/CT scans was similar to those described in previously reported studies^33,34^.

We assessed the impact on clinical management, of PT detection by PET/CT, by examining treatment decisions that were made by a referring physician or by a tumor board, and that were influenced by the identification or non-identification of PT. The performance of additional diagnostic procedures after a [18F]FDG-PET/CT study was not considered a change in management.

### Statistical analysis

Data are demonstrated as medians with ranges, or as means with standard deviations for continuous variables; and as percentages for categorical parameters. Correlations between subgroups were analyzed using the T-test for continuous variables, and the chi-square test and Fisher's exact test for categorical variables. The SPSS version 25.0 (SPSS, IBM, USA) was used. A P value of less than 0.05 was considered statistically significant.

### Ethics

The institutional review board of Sheba Medical Center approved our single-institution study, and informed consent was waived due to the retrospective design. All the methods were performed in accordance with the relevant guidelines and regulations of Sheba Medical Center.

## Data Availability

All data developed for and used in this study is available upon request of the authors.

## Abbreviations

CUP: Cancer of unknown primary site
PT: Primary tumor
SCC: Squamous cell carcinoma
PET: Positron emission tomography
[18F]FDG: 18-Fluorine-fluorodeoxyglucose
SUVmax: Maximum standardized uptake values
MIP: Maximum intensity projection
MRI: Magnetic resonance imaging
CT: Computed tomography
US: Ultrasound
